# Dietary Matrine Supplementation Enhances Growth, Immunity and Disease Resistance in Nile Tilapia (*Oreochromis niloticus*)

**DOI:** 10.3390/ani16101527

**Published:** 2026-05-16

**Authors:** Siyu Luo, Yue Liang, Chao Wang, Yifan Zhai, Xinyi Guo, Shupeng Zhang, Erlei Zhang, Dengfeng Yang, Du Guo, Tianqiang Liu, Gaoxue Wang, Erlong Wang

**Affiliations:** 1 Hainan Institute of Northwest A&F University, Sanya 572024, China; 2 Guangxi Key Laboratory of Marine Natural Products and Combinatorial Biosynthesis Chemistry, Guangxi Academy of Sciences, Nanning 530007, China; 3 College of Animal Science and Technology, Northwest A&F University, Yangling 712100, China; 4 College of Forestry, Northwest A&F University, Yangling 712100, China; 5 College of Information Engineering, Northwest A&F University, Yangling 712100, China; 6 Yulin Research Institute of Genuine Herbs of Qin Medicine, Yulin University, Yulin 719000, China; 7 Northwest A&F University Shenzhen Research Institute, Shenzhen 518000, China

**Keywords:** matrine, nile tilapia, growth performance, innate immunity, oxidative status

## Abstract

Matrine, a bioactive alkaloid from Sophora flavescens, has gained attention as a phytogenic additive due to its antioxidant and immunomodulatory properties. Nevertheless, Nile tilapia (*Oreochromis niloticus*) is highly vulnerable to *Streptococcus agalactiae* infection under intensive aquaculture, leading to immune dysfunction and economic losses. This study aimed to evaluate the potential application of dietary matrine supplementation in enhancing tilapia health, growth performance, and resistance to bacterial infection. Fish were randomly assigned to four dietary treatments: control, 0.1% matrine (low dose), 0.5% matrine (medium dose), and 1.0% matrine (high dose). Blood, liver, spleen, intestine, head kidney, and muscle were sampled on days 0, week 4, and week 8 for growth, immune, and oxidative stress analyses. Subsequently, fish were challenged with *S. agalactiae* to evaluate immune gene expression and liver histopathology across groups. The results showed that dietary matrine supplementation significantly improved growth performance and feed conversion efficiency, enhanced nonspecific immune responses, and increased antioxidant enzyme activities. Moreover, 0.5% and 1.0% matrine markedly reduced mortality and hepatic injury after bacterial challenge. These findings indicate that matrine acts as an effective natural immunomodulator and may contribute to hepato-intestinal health, and dietary supplementation at 1.0% represents the relatively favorable inclusion level under the present conditions for improving health status and supporting sustainable Nile tilapia aquaculture.

## Simple Summary

Farmed fish such as Nile tilapia are an important source of food worldwide, but intensive farming makes them more likely to suffer from bacterial diseases, leading to high losses and increased use of antibiotics. This study explored whether a natural plant compound called matrine, extracted from a traditional medicinal herb, could improve fish health in a safer and more sustainable way. Fish were fed diets containing different levels of this compound for eight weeks, and their growth, health status, and resistance to infection were evaluated. The results showed that fish receiving moderate amounts of matrine grew faster, used feed more efficiently, and had stronger natural defense systems and better protection against stress. After being exposed to harmful bacteria, these fish also had lower death rates and less damage to important organs. However, higher amounts did not provide additional benefits. Overall, the study suggests that adding an appropriate level of this natural compound to fish feed can improve animal health and reduce reliance on antibiotics, supporting more sustainable aquaculture and safer food production for society.

## 1. Introduction

Nile tilapia (*Oreochromis niloticus*) is one of the most widely farmed freshwater fish species worldwide due to its rapid growth, high feed efficiency, and strong environmental adaptability. However, intensive aquaculture practices have made tilapia increasingly susceptible to infectious diseases, among which *Streptococcus agalactiae* is one of the most prevalent and devastating pathogens. Infection with *S. agalactiae* often results in streptococcosis, characterized by systemic inflammation, neurological symptoms, and high mortality, leading to substantial economic losses. Current preventive measures primarily rely on antibiotics and vaccines, but these strategies are limited by the development of antibiotic resistance, poor vaccine efficacy in early developmental stages, and environmental safety concerns.

In recent years, natural herbal extracts have attracted increasing interest as sustainable and effective alternatives to conventional antimicrobial agents in aquaculture [1,2,3,4,5]. Among these, matrine—an active alkaloid extracted from Sophora flavescens—has been widely utilized in pharmacy, agronomy, and other fields [6]. The pharmacological activities of matrine are primarily attributed to its multiple biological functions, including antioxidant, immunomodulatory, antiviral, and antibacterial effects [7,8,9,10]. It has been shown to be effective in treating primary and metastatic breast cancer in mice [11], but its potential functional applications in farmed aquatic species such as Nile tilapia remain under investigation.

Preliminary studies in aquatic animals have revealed the hepatoprotective and immunostimulatory potential of herbal extracts under chemically or environmentally induced stress conditions [12,13,14]. However, a comprehensive evaluation of its effects on growth performance, immune capacity and antioxidant status in Nile tilapia is still lacking, especially under pathogenic challenge.

Therefore, the present study aimed to evaluate the effects of dietary matrine supplementation at concentrations of 0.1%, 0.5%, and 1.0% on growth performance, innate immune activity, antioxidant and anti-inflammatory responses, intestinal microbiota modulation, and post-challenge immune responses against *Streptococcus agalactiae* in Nile tilapia. This research provides new insights into the potential application of matrine as a natural functional feed additive to enhance fish health, immunity, and disease resistance in sustainable aquaculture systems.

## 2. Materials and Methods

### 2.1. Materials and Reagents

Matrine (analytical grade, ≥98% purity) was purchased from Yuanye Bio-Technology Co., Ltd. (Shanghai, China) and stored at 4 °C under dark and dry conditions until use. The basal diet used in this study was a commercial tilapia feed purchased from Tongwei Agricultural Development Co., Ltd. (Chengdu, China), identified as “Tilapia grow-out extruded feed (No. 1051)”. The proximate composition and nutrient levels of the basal diet are shown in Table 1. Experimental diets were formulated by supplementing 0.1%, 0.5%, and 1.0% matrine to the basal feed. All diets were pelleted into 2 mm diameter granules, dried at 60 °C for 12 h in a thermostatic oven. All experimental diets were prepared from the same batch and stored at 4 °C under dark and dry conditions throughout the feeding trial.

The *Streptococcus agalactiae* strain used in this study was preserved in our laboratory at −80 °C with 20% glycerol. Before the challenge experiment, the bacteria were cultured in BHI broth (37 °C, 12 h with shaking), harvested by centrifugation (5000× *g*, 10 min), and resuspended in PBS to a final concentration of 1 × 10^8^ CFU/mL. Commercial assay kits for ACP, AKP, T-AOC, and lysozyme were obtained from Jiancheng Bioengineering Institute (Nanjing, China). Bouin’s fixative and hematoxylin and eosin staining reagents were purchased from Beyotime Biotechnology (Shanghai, China). Reagents for RNA extraction (TRIzol), reverse transcription, and real-time quantitative PCR (RT-qPCR) were obtained from TaKaRa (Dalian, China).

### 2.2. Fish and Treatments

Healthy Nile tilapia (mean weight: 1.71 ± 0.33 g, mean length: 4.77 ± 0.23 cm) at about 3 months of age were randomly distributed into four dietary treatment groups: control (0% matrine, control group), low-dose (0.1% matrine, LM group), medium-dose (0.5% matrine, MM group), and high-dose (1.0% matrine, HM group). Each treatment consisted of three replicates with 20 fish per tank. Fish were reared in a recirculating aquaculture system under standard water quality conditions (30 ± 2 °C, pH 6.8–7.6, dissolved oxygen > 6 mg/L) for 8 weeks.

### 2.3. Growth Performance

Body weight and length measurements were conducted at day 0, the 4th week, and the 8th week. Fish were fasted for 24 h. Five fish per group were randomly selected for analysis at each time point. Growth parameters were evaluated by Mean body weight (MBW, g), Mean body length (MBL, cm), Hepatosomatic index (HSI, %), Weight gain rate (WGR, %), Specific growth rate (SGR, %/d), and Condition factor (CF, %). Growth performance parameters were calculated using the following formulas:

$$MBW\;(g)=\frac{\mathrm{Total}\;\mathrm{body}\;\mathrm{weight}\;\mathrm{of}\;\mathrm{fish}}{\mathrm{Number}\;\mathrm{of}\;\mathrm{fish}}$$

$$MBL\;(cm)=\frac{\mathrm{Total}\;\mathrm{body}\;\mathrm{length}\;\mathrm{of}\;\mathrm{fish}}{\mathrm{Number}\;\mathrm{of}\;\mathrm{fish}}$$

$$HSI\;(\%)=\frac{\mathrm{Liver}\;\mathrm{weight}\;(g)}{\mathrm{Body}\;\mathrm{weight}\;(g)}\times 100$$

$$WGR\;(\%)=\frac{\mathrm{Final}\;\mathrm{body}\;\mathrm{weight}-\mathrm{Initial}\;\mathrm{body}\;\mathrm{weight}}{\mathrm{Initial}\;\mathrm{body}\;\mathrm{weight}}\times 100$$

$$SGR\;(\%/day)=\frac{ln(\mathrm{Final}\;\mathrm{body}\;\mathrm{weight})-ln(\mathrm{Initial}\;\mathrm{body}\;\mathrm{weight})}{\mathrm{Experimental}\;\mathrm{days}}\times 100$$

$$CF\;(\%)=\frac{\mathrm{Body}\;\mathrm{weight}\;(g)}{{\mathrm{Body}\;\mathrm{length}\;(\mathrm{cm}}^{3})}\times 100$$

### 2.4. Immune Performance

At day 0, week 4, and week 8, 5 fish per group were euthanized by cervical dislocation. Blood was collected from the caudal vein using sterile syringes, allowed to clot at room temperature for 2 h, and centrifuged (12,000 rpm, 10 min at 4 °C) to obtain serum. Aliquots were stored at −20 °C for subsequent analysis. Liver, spleen, head kidney, and muscle tissues were immediately preserved in 200 μL TRIzol reagent (Invitrogen), flash-frozen in liquid nitrogen, and stored at −80 °C. Total RNA was extracted using the TRIzol-chloroform method. The integrity of the extracted RNA was verified by agarose gel electrophoresis, which displayed clear bands corresponding to 18S rRNA, 28S rRNA, and 5S rRNA, with the intensity of the 28S rRNA band being approximately twice that of the 18S rRNA. RNA purity was assessed using a spectrophotometer (Epoch, BioTek, Minneapolis, MN, USA), with OD 260/280 ratios between 2.0 and 2.2. Genomic DNA removal and cDNA synthesis were performed using the All-in-One First-Strand Synthesis MasterMix (Bestenzymes, Lianyungang, China). The resulting cDNA was detected by standard spectrophotometric methods using a spectrophotometer (Epoch, BioTek, USA) (OD 260/280 = 1.8–2.0) and adjusted to a uniform concentration of 100 ng/μL for subsequent applications.

### 2.5. Post-Challenge Analysis

At the end of the 8-week feeding trial, all experimental groups were challenged with *Streptococcus agalactiae* (1 × 10^8^ CFU/mL). At 24 h post-challenge, 5 fish per group were randomly selected. Spleen, head kidney and brain tissues were aseptically collected. Brain tissues were homogenized (1:9, *w*/*v*, in ice-cold PBS), serially diluted, and plated on BHI agar. After incubation at 37 °C for 24 h, photographs of the plates were taken, and colony numbers were counted using ImageJ (v1.54) software to calculate the bacterial load (CFU/g tissue). Spleen and head kidney were collected for RT-qPCR detection.

### 2.6. RT-qPCR

To explore the transcriptional levels of antioxidant-related genes, growth-related genes and immune-related genes, total RNA was extracted from muscle, spleen, liver and head kidney using the Trizol method and reverse-transcribed to cDNA. Prior to the bacterial challenge, muscle, spleen, and liver samples were collected for the analysis of immune-, antioxidant- and growth-related genes expression. Following the challenge, spleen and head kidney tissues were sampled to evaluate the expression levels of immune-related genes. qPCR was performed on a Bio-Rad CFX96 system using SYBR Green chemistry and the following primers (Table 2). Cycling conditions were: 95 °C for 30 s, followed by 40 cycles of 95 °C for 10 s and 60 °C for 30 s. Relative expression was calculated using the 2^−ΔΔCt^ method.

### 2.7. Histological Evaluation

Spleen, intestine, muscle, and liver tissues were fixed in formalin for 24 h, dehydrated in graded ethanol, embedded in paraffin, sectioned at 5 µm, and stained with hematoxylin and eosin (H&E). Histological changes including necrosis, inflammation, and hepatocyte integrity were evaluated under light microscopy.

### 2.8. Statistical Analysis

All data are expressed as Mean ± SE (Standard error). Statistical significance was analyzed using SPSS version 26. The *t*-test was utilized to compare two groups. Growth performance, serum biochemical indices, enzyme activities, bacterial load, and gene expression data involving different dietary treatments and sampling time points were mainly analyzed using two-way ANOVA. For multiple data groups, we first assessed data normality and homogeneity of variances using the Shapiro–Wilk and Levene’s tests, respectively. When data were normally distributed and homogenous, two-way ANOVA followed by Duncan’s test was performed. Alternatively, when the assumptions of normality or homogeneity of variance were not satisfied, the Kruskal–Wallis test was employed for multiple comparisons [15]. *p*-value < 0.05 was considered statistically significant.

## 3. Results

### 3.1. Effect of Matrine on Growth Performance

**Figure 1:**
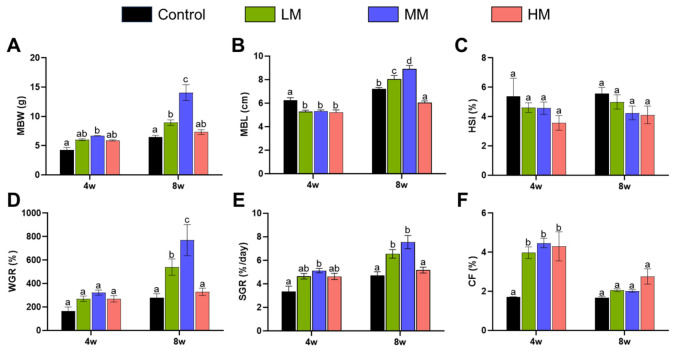
Effects of matrine on growth-related parameters of Nile tilapia. (**A**) Mean body weight (MBW, g), (**B**) Mean body length (MBL, cm), (**C**) Hepatosomatic index (HSI, %), (**D**) Weight gain rate (WGR, %), (**E**) Specific growth rate (SGR, %/d), (**F**) Condition factor (CF, %) of Nile tilapia fed with different concentrations of Matrine Control (0%), LM (0.1%), MM (0.5%) and HM (1.0%) at week 4 and week 8. Values are expressed as mean ± SE (*n* = 5). Different letters above bars indicate significant differences among groups at the same time point (*p* < 0.05).

Dietary supplementation with matrine markedly influenced the growth performance of Nile tilapia during the feeding trial (Figure 1). At week 8, fish fed diets containing 0.1% and 0.5% matrine exhibited significantly higher mean body weight (MBW), mean body length (MBL), weight gain rate (WGR), and specific growth rate (SGR) compared with the control group (*p* < 0.05). In contrast, the 1.0% matrine group showed growth performance comparable to that of the control at week 8, with no significant improvement observed in most parameters; notably, MBL in this group was even slightly lower than that of the control (Figure 1A,B,D,E). Throughout the experimental period, the hepatosomatic index (HSI) of all matrine-treated groups remained consistently lower than that of the control group (Figure 1C). Interestingly, dietary matrine supplementation significantly increased the condition factor (CF) of fish at week 4; however, no significant differences in CF were detected among groups at week 8 (Figure 1F).

Overall, these results indicate that matrine supplementation exerts a positive effect on growth performance in Nile tilapia in a dose- and time-dependent manner, with moderate supplementation levels conferring more pronounced and stable benefits than the highest dose.

### 3.2. Effect of Matrine on Antioxidative Status and Immune Performance in Different Tissues

**Figure 2:**
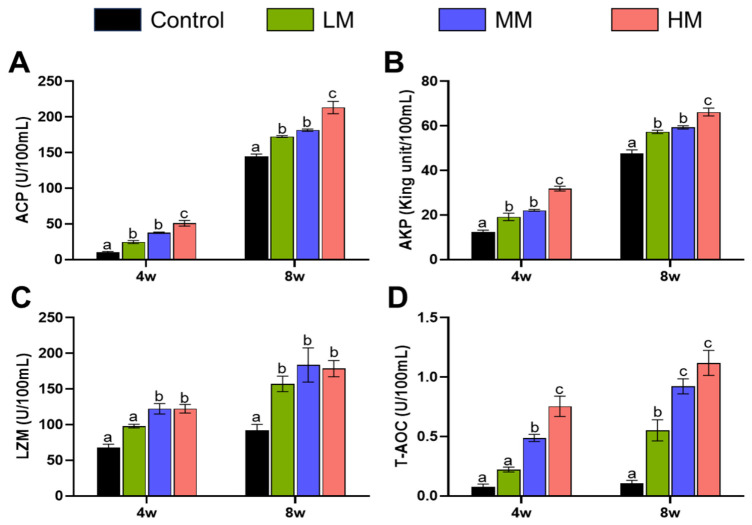
Effects of matrine on serum nonspecific immune and antioxidant indicators in Nile tilapia. (**A**) Acid phosphatase (ACP), (**B**) Alkaline phosphatase (AKP), (**C**) Lysozyme (LZM), (**D**) Total antioxidant capacity (T-AOC) levels in the serum of Nile tilapia fed diets supplemented with Control (0%), LM (0.1%), MM (0.5%) and HM (1.0%) matrine for 4 and 8 weeks. Data are presented as mean ± SE (*n* = 5). Different letters above bars indicate significant differences among groups at the same time point (*p* < 0.05).

Dietary matrine supplementation significantly influenced serum enzyme activities related to innate immunity and antioxidant capacity in Nile tilapia (Figure 2). As shown in Figure 2A, serum acid phosphatase (ACP) activity was markedly elevated in all matrine-treated groups compared with the control at week 4 (*p* < 0.05), with no significant difference detected between the 0.1% and 0.5% supplementation levels. By week 8, ACP activity remained significantly higher in all matrine-supplemented groups than in the control (*p* < 0.05), while the difference between the 0.1% and 0.5% groups was still not significant. A similar enhancement pattern was observed for alkaline phosphatase (AKP). At both week 4 and week 8, all matrine-treated groups exhibited significantly increased AKP activity relative to the control group (*p* < 0.05). However, no significant difference was found between the 0.1% and 0.5% matrine groups at either sampling point (Figure 2B). Lysozyme (LZM) activity showed a more differentiated response among treatments. At week 4, the 0.1% matrine group did not differ significantly from the control, whereas both the 0.5% and 1.0% groups displayed significantly higher LZM activity (*p* < 0.05), with no significant difference between these two higher-dose groups. By week 8, LZM activity was significantly elevated in all matrine-supplemented groups compared with the control, while no significant differences were observed among the treatment groups (Figure 2C). Regarding total antioxidant capacity (T-AOC), no significant difference was detected between the 0.1% matrine group and the control at week 4. In contrast, T-AOC levels in the 0.5% and 1.0% groups were significantly higher than those of the control, and a significant difference was also observed between these two groups at this time point. At week 8, all matrine-treated groups exhibited significantly increased T-AOC compared with the control; however, the difference between the 0.5% and 1.0% groups was no longer significant (Figure 2D).

Overall, serum enzyme activities associated with innate immunity and antioxidant defense were generally enhanced by dietary matrine supplementation. Although the responses of individual parameters varied with time and enzyme type, the magnitude of enhancement tended to increase with increasing matrine concentration, suggesting a potential dose-related trend in serum enzyme activity regulation.

### 3.3. Effects of Matrine on Immune, Growth, and Antioxidant-Related Genes Expression in Nile Tilapia

**Figure 3:**
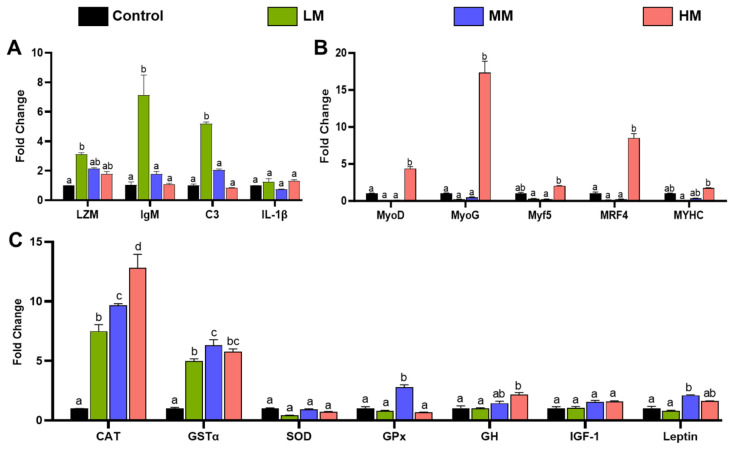
Effects of dietary matrine supplementation on immune-, growth-, and antioxidant-related gene expression in Nile tilapia. (**A**) Immune-related genes (LZM, IgM, C3, and IL-1β) in the spleen, (**B**) growth-related genes (MyoD, MyoG, MRF4, Myf5, and MYHC) in the muscle, and (**C**) antioxidant- and metabolism-related genes (CAT, GSTα, SOD, GPx, GH, IGF-1, and Leptin) in the liver of Nile tilapia fed diets containing Control (0%), LM (0.1%), MM (0.5%) and HM (1.0%) matrine for 8 weeks. Gene expression levels were normalized to EF1α and analyzed using the 2^−ΔΔCt^ method. Data are presented as mean ± SE (*n* = 5). Different letters above bars indicate statistically significant differences among groups (*p* < 0.05).

Figure 3 presents the relative expression levels of immune, growth, and antioxidant-related genes in Nile tilapia fed diets supplemented with different concentrations of matrine. As shown in Figure 3A, the expression levels of lysozyme (LZM), immunoglobulin M (IgM), and complement component 3 (C3) were significantly upregulated in the 0.1% matrine group compared with the control (*p* < 0.05), while no significant differences were observed in the 0.5% and 1.0% groups. The expression of interleukin-1β (IL-1β) showed no significant variation among all treatments, suggesting that matrine primarily modulated humoral immune factors rather than inducing pro-inflammatory cytokine activation at this stage.

Dietary matrine had a notable effect on growth-related gene expression. The 1.0% matrine group exhibited significantly higher transcription levels of MyoD, MyoG, and MRF4 compared with the control (*p* < 0.05), indicating enhanced muscle differentiation and development. However, no significant differences were detected among treatments for Myf5 and MYHC, suggesting that these genes were less responsive to matrine supplementation under the present conditions (Figure 3B).

Figure 3C shows the expression profiles of antioxidant- and metabolism-related genes. The expression of catalase (CAT) and glutathione S-transferase alpha (GSTα) was significantly elevated in all matrine-treated groups (*p* < 0.05), with the highest CAT expression observed in the 1.0% group and the strongest GSTα induction in the 0.5% group. For glutathione peroxidase (GPx) and leptin, significant upregulation occurred only in the 0.5% matrine group compared to the control (*p* < 0.05). In contrast, growth hormone (GH) expression increased significantly only in the 1.0% group, while no significant differences were detected among treatments for superoxide dismutase (SOD) or insulin-like growth factor-1 (IGF-1).

### 3.4. Effect of Matrine on the Histological Structure of Different Tissues in Nile Tilapia

**Figure 4:**
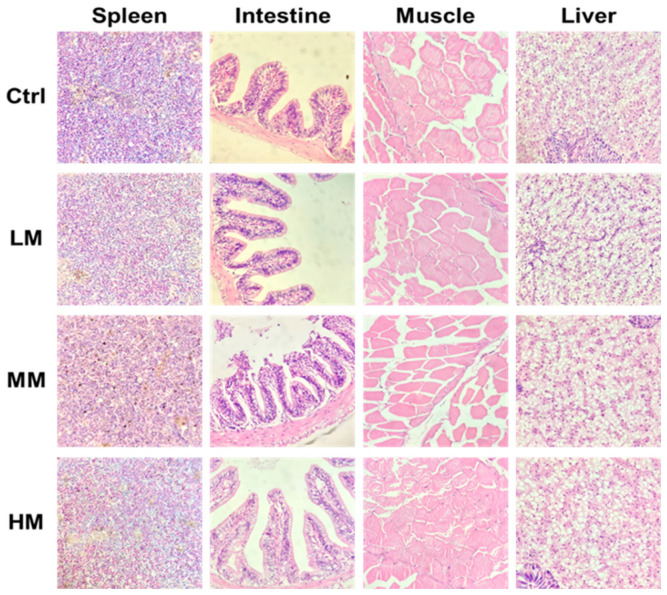
Histological sections of spleen, intestine, muscle, and liver of Nile tilapia fed diets with different concentrations of matrine before challenge. Histological sections of spleen, intestine, muscle, and liver from Nile tilapia fed diets supplemented with different concentrations of matrine were stained with hematoxylin and eosin (H&E) and observed under a light microscope (40× magnification).

Histological observations of spleen, intestine, muscle, and liver tissues from Nile tilapia before pathogen challenge are shown in Figure 4. The structural integrity of all tissues remained normal across treatments, indicating that dietary matrine supplementation at experimental concentrations did not induce overt pathological alterations. However, distinct morphological variations were observed among different groups, reflecting the physiological modulation of growth and metabolism by matrine.

In the spleen, brown pigment aggregations were observed in all groups. These pigment deposits are generally associated with erythrocyte degradation and immune–metabolic processes. The 0.5% matrine group showed a relatively moderate distribution of melanomacrophage centers (MMCs) with a more uniform appearance, which may indicate a relatively stable immune–metabolic status and efficient tissue turnover, consistent with the observed improvements in growth performance. In contrast, the more pronounced pigment accumulation observed in the 1.0% group may suggest a slight increase in oxidative or metabolic stress, possibly reflecting elevated detoxification demand at high matrine levels.

The intestinal morphology revealed clear differences in villus structure and mucosal organization. Fish fed with 0.5% matrine showed the most compact mucosal folds and thickened intestinal walls, indicative of enhanced absorptive capacity and epithelial health. The 1.0% group displayed longer villi but with notable intracellular lipid droplets, suggesting that although the absorptive surface increased, excessive matrine may have promoted lipid accumulation beyond the metabolic capacity of the fish. The slight disruption observed in some villi of the 0.5% group was likely due to mechanical artifacts during sectioning rather than actual tissue damage.

In muscle sections, clear distinctions were also observed among different treatment groups. The control group exhibited regularly aligned myofibers with narrow intercellular spaces, representing normal muscular development. The 0.1% group showed a compact arrangement similar to the control, while the 0.5% matrine group presented the most well-organized myofibrillar structure with densely packed fibers and minimal interstitial gaps, reflecting enhanced protein deposition and muscle growth efficiency. In contrast, fish fed with 1.0% matrine displayed slightly loosened muscle fiber organization and occasional intracellular vacuoles, suggesting mild myofibrillar degeneration or metabolic stress possibly caused by excessive matrine intake. These findings further corroborate that moderate matrine supplementation supports optimal muscular development and overall growth in Nile tilapia.

In the liver, hepatocytes of all groups were arranged regularly without significant necrosis or degeneration, demonstrating normal hepatic structure. However, the 1.0% matrine group exhibited more pronounced cytoplasmic vacuolation, indicating possible lipid deposition or early-stage steatosis. This observation, combined with the intestinal findings, suggests that high matrine inclusion might lead to metabolic burden, reducing its growth-promoting efficacy. The 0.5% matrine group displayed relatively intact hepatic structure, with well-defined cell boundaries and minimal vacuolation.

### 3.5. Effect of Matrine on Bacteria Loading and Immune Performance in Different Tissues of Nile Tilapia Post-Challenge

**Figure 5:**
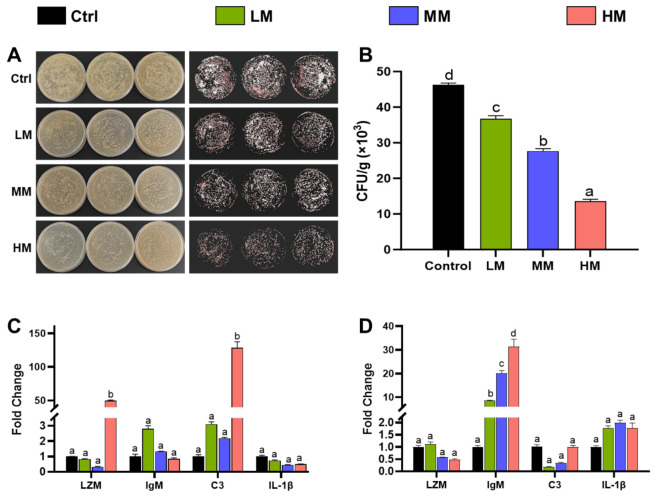
Effects of dietary matrine supplementation on bacterial loading and immune-related gene expression in different tissues of Nile tilapia post-challenge. (**A**). Plate colony count of brain samples under different dietary Matrine concentrations; (**B**). Bacterial load (CFU/g) in the brain of Nile tilapia fed diets containing Control (0%), LM (0.1%), MM (0.5%) and HM (1.0%) Matrine; (**C**). Relative mRNA expression levels of immune-related genes (LZM, C3, IgM, and IL-1β) in the spleen after bacterial challenge; (**D**). Relative mRNA expression levels of immune-related genes (LZM, C3, IgM, and IL-1β) in the head kidney after bacterial challenge. Gene expression levels were normalized to EF1α and analyzed using the 2^−ΔΔCt^ method. Data are presented as mean ± SE (*n* = 5). Different letters above bars indicate statistically significant differences among groups (*p* < 0.05).

As shown in Figure 5, the bacterial load in the brain and the expression of immune-related genes in the spleen and head kidney were evaluated after *Streptococcus agalactiae* challenge under different concentrations of matrine treatment. Figure 5A,B illustrated that bacterial loads in the brain significantly decreased with increasing concentrations of matrine, and the differences among all treatment groups were statistically significant (*p* < 0.05). In the spleen (Figure 5C), the expression levels of LZM and C3 genes were markedly upregulated in the 1.0% matrine-treated group compared with the control, whereas no significant differences were observed in other treatment groups. For IgM and IL-1β, no significant variation was detected among all treatments, suggesting that matrine may selectively modulate specific immune genes in the spleen. In the head kidney (Figure 5D), matrine supplementation notably enhanced IgM gene expression across all treated groups, and the upregulation trend was positively correlated with matrine concentration. However, the expressions of LZM, C3, and IL-1β did not show significant differences among groups, indicating that matrine exerts its primary immunostimulatory effect in the head kidney through the activation of IgM expression.

## 4. Discussion

The present study systematically evaluated the effects of dietary matrine supplementation on growth performance, immune and antioxidant status, gene expression profiles, tissue histology, and resistance against *Streptococcus agalactiae* infection in Nile tilapia (*Oreochromis niloticus*). Overall, the findings demonstrate that matrine functions as a phytogenic feed additive capable of enhancing host physiological performance and disease resistance, with moderate dietary inclusion (1.0%) providing the most consistent benefits across multiple biological levels.

Dietary matrine supplementation significantly improved growth-related parameters, including MBW, MBL, WGR, and SGR, particularly at 0.1% and 0.5% inclusion levels by week 8. In contrast, the highest supplementation level (1.0%) did not further enhance growth and, in some parameters, exhibited values comparable to the control group. This pattern suggests a dose-dependent but non-linear growth response, a phenomenon frequently reported for phytogenic feed additives in tilapia and other teleost species [16,17]. The early increase in condition factor (CF) observed at week 4, followed by convergence among treatments at week 8, indicates that matrine may initially promote energy deposition and somatic condition, whereas long-term growth regulation tends to stabilize body proportionality. Similar temporal responses have been described in tilapia fed plant-derived bioactive compounds, where early metabolic stimulation does not necessarily result in sustained differences at later stages [18]. Moreover, the consistently lower hepatosomatic index (HSI) in matrine-supplemented groups may be associated with altered metabolic status and reduced lipid accumulation, which may partly explain the enhanced growth performance observed at optimal inclusion levels.

Innate immune parameters and antioxidant enzyme activities were markedly influenced by matrine supplementation. The significant elevation of ACP, AKP, LZM, and T-AOC activities indicates an overall enhancement of non-specific immune defense and oxidative stress resistance. These effects were evident at both sampling points, with stronger responses generally observed at medium and high doses during the early feeding stage. Such immunostimulatory effects are consistent with previous studies demonstrating that phytogenic additives stimulate lysosomal enzyme activity, enhance macrophage function, and improve redox balance in tilapia [5,19]. The progressive increase in serum enzyme activities with increasing matrine concentration suggests a dose-responsive trend; however, excessive inclusion did not necessarily confer superior long-term advantages. Improved antioxidant capacity likely contributes to immune homeostasis by limiting oxidative damage during immune activation, thereby supporting sustained physiological performance.

At the molecular level, matrine supplementation significantly modulated the expression of genes associated with immunity (e.g., LZM, C3, IgM), antioxidant defense (CAT, GSTα), and growth regulation (MyoD, MyoG). Moderate supplementation levels induced coordinated upregulation of immune- and antioxidant-related genes across multiple tissues, indicating systemic activation of protective pathways. These findings are in agreement with previous reports showing that dietary plant extracts and phytochemicals regulate immune and antioxidant gene networks in Nile tilapia, thereby enhancing host resilience to environmental and pathogenic stressors [18,20]. Interestingly, higher matrine concentrations selectively promoted growth-related gene expression in muscle tissue without corresponding improvements in growth performance, suggesting that excessive supplementation may trigger compensatory transcriptional responses rather than functional gains. This observation underscores the importance of identifying an optimal dosage to maximize physiological efficiency while avoiding unnecessary metabolic burden.

Histological examination further supported the functional advantages of moderate matrine supplementation. The improved intestinal villus architecture and increased mucosal thickness observed in the 0.5% group indicate enhanced nutrient absorption capacity, which likely contributed to superior growth outcomes. Comparable improvements in gut morphology have been reported in tilapia fed phytogenic additives, where intestinal integrity is closely linked to feed utilization efficiency [21]. In the liver, moderate matrine inclusion maintained normal hepatocyte organization, whereas higher doses showed increased vacuolization, possibly reflecting altered lipid metabolism. The presence of pigment accumulation in the spleen across treatments likely represents physiological melanomacrophage activity rather than pathological alteration and may be associated with immune activation rather than impaired growth. Muscle histology exhibited a dose-dependent pattern, with optimal supplementation maintaining compact fiber structure, whereas excessive inclusion resulted in mild fiber loosening and vacuolation, suggesting suboptimal tissue organization at higher inclusion levels.

Following bacterial challenge, matrine-supplemented fish exhibited reduced bacterial load in brain tissue and enhanced expression of immune-related genes in spleen and head kidney, particularly in the 1.0% group. These results indicate that matrine strengthens systemic immune responsiveness and pathogen clearance capacity. Comparable outcomes have been reported for phytogenic blends and plant extracts that improved survival and immune gene activation in tilapia challenged with *S. agalactiae* or Aeromonas hydrophila [16,20]. In addition, previous studies have demonstrated that matrine could enhance resistance against White Spot Syndrome Virus (WSSV) infection in crayfish, further supporting the antimicrobial and immunoprotective potential of matrine in aquatic animals [22]. Similar antiviral effects of matrine have also been reported in fish viral infection models, where matrine significantly inhibited Singapore grouper iridovirus (SGIV) replication by regulating inflammatory responses, antioxidant activity, and interferon-related pathways, particularly during the early stage of infection [23]. Furthermore, dietary supplementation with phytogenic compounds containing matrine-like bioactive alkaloids has been shown to improve immune responses, antioxidant capacity, and disease resistance in aquatic animals, suggesting that plant-derived immunostimulants may exert broad-spectrum protective effects against pathogenic stress in aquaculture species [24]. The combined enhancement of innate immune enzymes, antioxidant defenses, immune gene expression, and tissue integrity likely underlies the improved post-challenge performance observed in matrine-supplemented fish. Collectively, these results suggest that matrine may contribute to improved resistance through a potential multi-level immunomodulatory effect rather than acting on a single immune pathway, a pattern that has also been reported in other fish species receiving plant-derived bioactive compounds [25].

Taken together, the present findings indicate that dietary matrine supplementation exerts beneficial effects on growth performance, immune competence, antioxidant capacity, tissue integrity, and bacterial resistance in Nile tilapia. Among the tested levels, the 1.0% matrine group shows more consistent improvements across physiological, immune-, and antioxidant-related gene expression, suggesting that this inclusion level may be more favorable for maintaining overall performance under the present experimental conditions.

In addition, the 1.0% supplementation level exhibited the most pronounced inhibitory effect on *Streptococcus agalactiae* proliferation following challenge, as reflected by the lowest bacterial load. However, since sampling was conducted at 24 h post-challenge, this result may primarily reflect an early-stage inhibitory effect of high-dose matrine on bacterial infection. Therefore, the long-term protective efficacy of matrine against *S. agalactiae* remains to be further clarified.

Overall, these findings support the potential application of matrine as a phytogenic feed additive in tilapia aquaculture while highlighting the importance of dosage considerations depending on specific functional objectives. In addition, phytogenic feed additives and herbal-derived bioactive compounds have been increasingly recognized as economically valuable alternatives in aquaculture due to their abilities to improve feed utilization, enhance disease resistance, reduce dependence on chemotherapeutics, and potentially lower production losses associated with intensive farming systems [26,27,28]. Previous studies have suggested that plant-derived additives, including alkaloids and herbal extracts, may contribute to improved growth efficiency and sustainable aquaculture practices with relatively low environmental impact [29]. Therefore, although purified matrine showed promising biological effects in the present study, further comparative evaluations of the cost–benefit efficiency between purified matrine and whole herbal additives would still be valuable for large-scale practical applications.

## 5. Conclusions

In summary, dietary matrine supplementation was associated with improvements in growth performance, innate immunity, antioxidant capacity, tissue condition, and resistance to *Streptococcus agalactiae* infection in Nile tilapia. Among the tested levels, the 1.0% matrine supplementation tended to yield more consistent responses across physiological, molecular, histological, and post-challenge assessments, whereas higher supplementation did not appear to confer proportional benefits. The results further suggest that matrine may promote fish health through coordinated modulation of growth, immune defense, and oxidative status, rather than acting on a single pathway. Therefore, under the present experimental conditions, a dietary supplementation level around 1.0% may represent a more favorable option, although this requires further validation. These findings provide supporting evidence for the potential use of matrine as a phytogenic feed additive and may contribute to the development of more sustainable and antibiotic-reduced tilapia aquaculture strategies.

## Figures and Tables

**Table 1:** Proximate composition of the basal diet.

Component	Content (%)
Crude protein (≥)	30.0
Crude lipid (≥)	6.0
Crude fiber (≤)	12.0
Ash (≤)	15.0
Calcium	0.5–2.0
Total phosphorus (≥)	0.8
Moisture (≤)	11.0
Lysine (≥)	1.4

**Table 2:** Primers used in this study.

Genes	Primers Sequence (5′-3′)	Resources
*CAT*	F: ACATGCCACCAGGAATCGAG	XM_003447521
	R: ATCTGCAGGTAGTTTGCCCC	
*GSTα*	F: TAATGGGAGAGGGAAGATGG	NM_001279635.1
	R: CTCTGCGATGTAATTCAGGA	
*SOD*	F: CGCCTTTTACAGATGACCAT	XM_003454189.4
	R: GTGTCGCTGGATGCTAAGA	
*GPx*	F: GTGCCCTGCAATCAGTTTGG	NM_001279711.1
	R: CGAGGAGCTGGAACTTTGGT	
*GH*	F: GTTGTGTGTTTGGGCGTCTC	HM_565014.1
	R: CAGGTGCGTGACTCTGTTGA	
*IGF-1*	F: TCCTGTAGCCACACCCTCTC	NM_001279503.1
	R: ACAGCTTTGGAAGCAGCACT	
*Leptin*	F: GAAGTGGATCGCTGAGCATCTGG	XM_005449522.4
	R: CCATCCAAGCAGACCGTGACTATG	
*MyoD*	F: CCACCTGTCAGACAACCAGA	GU246722
	R: ACTGCGTTCGCTCTTCAGAC	
*MyoG*	F: CTCAACCAGCAGGACACTGA	GU246725
	R: ATCCTCGCTGCTGTAGCTCT	
*Myf5*	F: CTGCTCTGATGGCATGGCTGA	XM_005456634
	R: CACGATACTGGACAGGCACTC	
*MRF4*	F: TGGCAATGACAGCCCACTG	PQ497691
	R: CTTACGTCTATCCGTGGGAG	
*MYHC*	F: GGAAGGCATCGAGTGGGA	XM_013273306.3
	R: CTGGTAGAGTTGGACGACAGTTT	
*LZM*	F: AAGGGAAGCAGCAGCAGTTGTG	XM_003460550.2
	R: CGTCCATGCCGTTAGCCTTGAG	
*IgM*	F: ACCGAATCGAAAAATGCGGC	KJ676389.1
	R: AACACAACCAGGACATTGGTTC	
*C3*	F: GGTGTGGATGCACCTGAGAA	XM_013274267.2
	R: GGGAAATCGGTACTTGGCCT	
*IL-1β*	F: GACACGATGCGATTCCTATTCT	XM_019365841.2
	R: CACTGGGCAGTCTTCTCGGA	
*EF1α*	F: TGATCTACAAGTGCGGAGGAA	AB075952.1
	R: GGAGCCCTTTCCCATCTCA	

## Data Availability

All data used in this study are presented in this article.

## Abbreviations

The following abbreviations are used in this manuscript:

**Abbreviation**	**Full term**
ACP	Acid phosphatase
AKP	Alkaline phosphatase
CAT	Catalase
CF	Condition factor
GPx	Glutathione peroxidase
HSI	Hepatosomatic index
IgM	Immunoglobulin M
LZM	Lysozyme
MBW	Mean body weight
MBL	Mean body length
MMCs	Melanomacrophage centers
SGR	Specific growth rate
SOD	Superoxide dismutase
T-AOC	Total antioxidant capacity
WGR	Weight gain rate
